# Managing Bacteremia: Insights Into Pathogen-Specific Treatment

**DOI:** 10.7759/cureus.78674

**Published:** 2025-02-07

**Authors:** Mariana Salvado de Morais, Ana Gonçalves, Gonçalo Cristóvão, José Lucena, Ana Catarina Reis, Stanislav Tsisar, Sofia Ramos, Mario Rodrigues

**Affiliations:** 1 Internal Medicine, Centro Hospitalar Universitário Lisboa Central, Lisbon, PRT; 2 Infectious Diseases, Centro Hospitalar Universitário Lisboa Central, Lisbon, PRT; 3 Critical Care, Centro Hospitalar Universitário Lisboa Central, Lisbon, PRT

**Keywords:** bacteremia, coagulase-negative staphylococci, enterobacterales, enterococcus, gram-negative bacteremia, gram-positive bacteremia, mrsa bacteremia, mssa bacteremia, pseudomonas aeruginosa, staphylococcus aureus

## Abstract

Recent advances in the treatment of bacteremia have challenged traditional approaches, particularly regarding the duration of antibiotic therapy and the transition from intravenous to oral regimens. This paper reviews these updates, focusing on evidence-based strategies for managing bacteremia caused by Gram-positive and Gram-negative organisms. Criteria for transitioning to oral therapy, clinical decision-making for uncomplicated cases, and evidence supporting shorter antibiotic courses are discussed. This article is a narrative review of the current literature, integrating clinical guidelines, trial data, and specific considerations for diverse pathogens, ensuring a comprehensive perspective.

## Introduction and background

Bacteremia, defined as the presence of bacteria in the bloodstream, poses a significant clinical challenge due to its association with high morbidity and mortality [1]. If left untreated or inadequately managed, bacteremia can progress to sepsis, a life-threatening condition that may lead to organ failure and shock. Additionally, bacteria in the bloodstream can spread to distant organs, resulting in metastatic infections, such as infective endocarditis (IE), osteomyelitis, or abscess formation.

The management of bacteremia has traditionally relied on prolonged intravenous (IV) antibiotic therapy, which ensures reliable drug delivery but also presents significant challenges. Prolonged IV treatment increases the risk of catheter-associated bloodstream infections, a serious complication that occurs when bacteria colonize the IV catheter, leading to further bloodstream infections. Extended hospital stays required for IV therapy also expose patients to hospital-acquired infections, reduce mobility, and negatively impact quality of life. Furthermore, the high resource utilization associated with prolonged hospitalization contributes to increased healthcare costs [2]. 

Recent developments have called for a re-evaluation of these practices. Evidence now supports shorter antibiotic courses and transitions to oral therapy in select patients, provided they meet specific clinical criteria. These approaches have been shown to reduce healthcare burdens without compromising patient outcomes [2]. This paper reviews current evidence and practice updates, emphasizing the importance of individualized treatment plans based on the pathogen type, patient condition, and clinical circumstances.

This article is a narrative review of the current literature, integrating clinical guidelines, and synthesizes findings from pivotal clinical trials, practice guidelines, and pharmacoeconomic studies to present a comprehensive overview of bacteremia management. The treatment of bacteremia caused by the pathogens, *Staphylococcus aureus*, coagulase-negative *Staphylococci*, *Enterococcus* species, *Enterobacterales*, and *Pseudomonas aeruginosa, *will be discussed. Particular focus is given to the criteria for transitioning from IV to oral therapy, the selection of antibiotics based on pathogen profiles, and the duration of treatment. The study includes references to landmark studies such as the SABATO trial, IDSA guidelines, and trials comparing short-course *versus *standard-course therapy for both Gram-positive and Gram-negative infections [3-6]. 

## Review

Bacteremia is a serious medical condition that can lead to severe complications, including endocarditis, spondylodiscitis, sepsis, and organ failure. The treatment of bacteremia focuses on eliminating the underlying infection, managing symptoms, and preventing potential complications. This condition is notable due to its rapid progression and the critical need for timely medical intervention to improve patient outcomes. Various therapeutic approaches, including antibiotic therapy, supportive care, and surgical intervention, are employed to address this complex infection effectively. Antibiotic therapy is the cornerstone of bacteremia treatment, with the initial use of broad-spectrum antibiotics followed by targeted therapy based on blood culture (BC) results. In certain cases, surgical procedures may be necessary to remove the source of infection, such as abscesses or prosthetic device infection, especially when antibiotics alone are insufficient [1,2]. 

In recent years, new approaches that categorize patients as 'high risk' or 'low risk' for metastatic infection based on clinical, microbiological, and imaging data have emerged. ‘High risk’ factors include persistent fever despite appropriate antibiotic therapy, immunosuppression, presence of indwelling medical devices, history of recent surgery, presence of structural heart disease or prior endocarditis, symptoms suggestive of a metastatic focus, and evidence of organ dysfunction or sepsis and the presence of high-risk pathogens. Preventive measures are critical in reducing the incidence of bacteremia, encompassing practices such as maintaining oral hygiene and implementing stringent infection control protocols in healthcare environments [7].

This article aims to provide practical clinical guidance for clinicians seeking to enhance their management of bacteremia. The collected evidence was critically analyzed and summarized to provide a comprehensive overview of current knowledge. A systematic approach was employed to explore key databases, selecting studies based on their relevance, methodological quality, and contribution to the topic. Specifically, a search was conducted on PubMed using the keywords "bacteremia," "gram-positive", "gram-negative", “*Staphylococcus aureus* bacteremia”, “Coagulase-Negative *Staphylococci *bacteremia”, “*Enterococcus* bacteremia” “*Enterobacterales* bacteremia”, and “*Pseudomonas aeruginosa *bacteremia” focusing on studies published since 2020. The gathered evidence was critically analyzed and summarized to present a cohesive overview of current knowledge. While this review does not include a formal meta-analysis, it provides a structured synthesis of the most significant findings. Additionally, this narrative review aims to offer clinicians a reliable and comprehensive evaluation of the literature, including data from multicentric studies, to support informed decision-making and ultimately improve clinical outcomes. 

Antibiotic management for pathogens

The treatment of bacteremia caused by the pathogens, S*taphylococcus aureus, *Coagulase-negative Staphylococci, Enterococcus species, *Enterobacterales, and Pseudomonas aeruginosa, *will be discussed.

The selected pathogens were chosen for their clinical significance, therapeutic complexity, and public health relevance. These organisms represent some of the most prevalent and challenging causes of bacteremia, each requiring distinct management strategies. Addressing these pathogens provides a comprehensive understanding of core principles in bacteremia treatment, including the importance of tailored antimicrobial therapy and source control. 

Gram-positive organisms

Staphylococcus aureus

*Staphylococcus aureus* bacteremia (SAB) is associated with a high one-year mortality rate of approximately 30% [8]. This pathogen is rarely a contaminant, and its isolation, even from a single sample, warrants careful consideration. Due to its high morbidity and mortality, SAB requires vigilant attention from healthcare providers to ensure appropriate management [6]. A key feature of this infection is the increased risk of distant infection foci, such as endocarditis and spondylodiscitis. Consequently, a critical component of the treatment approach is the collection of multiple BCs after the initiation of antibiotic therapy to determine whether the bacteremia is complicated or uncomplicated. In recent years, several studies have shown that the first follow-up cultures should be performed after 24 hours, before the recommended interval of 48-72 hours, to help identify patients at increased risk of death and metastatic spread [3,4,8,9].

Although it is difficult to define uncomplicated bacteremia, it can be defined by several factors: the achievement of sterile BCs within two to four days after starting antibiotics, the resolution of fever within 72 hours of initiating treatment, the identification and removal of the infection's focus, the absence of heterologous materials (e.g., prosthetic devices or foreign bodies), and the lack of secondary hematogenous infection foci. Additionally, endocarditis must be excluded in nearly all patients, with this being performed within the first seven days after the first positive BC. If any of these criteria are not met, the bacteremia is classified as complicated [10].

The standard treatment for uncomplicated *Staphylococcus aureus* bloodstream (Table 1) infection involves antibiotic therapy for 14 days after negative BC. For methicillin-sensitive *Staphylococcus aureus *(MSSA), penicillin with anti-staphylococcal activity, such as oxacillin or flucloxacillin, or a first-generation cephalosporin such as cefazolin, is used, while methicillin-resistant *Staphylococcus aureus* (MRSA) is treated with vancomycin, daptomycin or linezolid [3,6,11-13].

**Table 1:** Management of Staphylococcus aureus bacteremia Source: [3,5,6,14] MSSA: Methicillin-sensitive Staphylococcus aureus; MRSA: methicillin-resistant Staphylococcus aureus

Pathogen	IV therapy options	Oral therapy options (if clinical criteria are met)
MSSA	Oxacillin 2g every 4h; flucloxacillin 2g every 6h; cefazolin 2g every 8h	Trimethoprim-sulfamethoxazole 160/800mg every 12h; clindamycin 600mg every 8h
MRSA	Vancomycin 15-20mg/kg every 8-12h; daptomycin 8-10mg/kg every 24h	Trimethoprim-sulfamethoxazole 160/800mg every 8-12h; linezolid 600mg every 12h

Duration: The duration for treatment is 14 days (minimum of 5-7 days IV). It is crucial to emphasize that the durations provided are dependent on positive clinical progress and should be personalized based on each patient's condition and response to treatment [3,6,15].

In recent years, several studies have demonstrated the efficacy and safety of transitioning from intravenous to oral antimicrobial therapy for patients with low-risk *Staphylococcus aureus* bloodstream infections. The SABATO trial included adults diagnosed with low-risk SAB after receiving 5-7 days of IV therapy. Afterward, participants were randomized to either oral antimicrobial therapy or continued IV therapy. All patients with complicated bacteremia were excluded, such as those with endocarditis, deep-seated infections, prosthetic devices, or severe comorbidities. The study demonstrated non-inferiority of oral antimicrobial therapy compared to IV therapy in low-risk SAB. The oral switch group showed no significant difference in SAB-related complications, suggesting that an early transition to oral therapy can be a safe and effective strategy for select patients. The shorter hospital stay observed in the oral group supports the benefits of oral therapy in reducing healthcare costs and improving patient quality of life by minimizing hospital-related risks and burdens [3,5,6].

This study offers strong evidence for adopting oral antimicrobial therapy in appropriately selected patients with SAB. Based on this study after 5-7 days of IV therapy, in selected patients, with a favorable evolution (apyrexia after 72 hours of therapy, negative BC and with certain uncomplicated bacteremia), a switch to oral antibiotics can be made, either with trimethoprim-sulfamethoxazole 160/800mg/8-12h (first choice), clindamycin 600mg/8h for MSSA; trimethoprim-sulfamethoxazole 160/800mg/8-12h (first choice) or linezolid 600mg 12h for MRSA [3,6]. The total antibiotic course has to be 14 days [6]. It is important to notice that the collaboration with infectious disease specialists and adherence to antimicrobial stewardship principles are essential for ensuring optimal outcomes [8,12,13].

Coagulase-Negative Staphylococci (CoNS)

CoNS are the most frequent isolates in BCs and are considered contaminants in approximately half of the cases, given their role as skin commensals. Although they are considered microorganisms of low virulence, their ability to adhere to biomaterials and produce biofilm makes them adept at surviving on various surfaces. Thus, when bacteremia is confirmed, targeted treatment should be considered, particularly in immunocompromised patients or individuals with prosthetic devices [1,9]. In an attempt to decrease the overuse of antibiotics and its consequences, recent studies have proposed an algorithm-based treatment which divides CoNS bacteremia into three groups: simple, uncomplicated, and complicated [12,14,16].

Simple CoNS bacteremia is considered when there is a single BC isolating said microorganism, a negative follow-up BC, no signs or symptoms of local or metastatic infection and absence of intravascular prosthetic devices. The rationale is that simple CoNS bacteremia typically represents transient bacteremia, often related to contamination during sample collection or a self-limiting process. Short-course antibiotic therapy is usually sufficient.

Uncomplicated bacteremia requires two or more BC isolating CoNS collected less than 24 hours apart or a single BC isolating said bacteria plus signs of local infection at the catheter site. Uncomplicated cases require a more extended course of antibiotics due to the presence of localized infection or the potential for bacterial persistence, even if complications like endocarditis or metastatic infections are not present.

Lastly, complicated bacteremia is defined as two or more positive BCs drawn more than 24 hours apart, with antibiotic therapy, or signs of metastatic infection (e.g., endocarditis documented on echocardiography). It indicates more severe infection with evidence of systemic or localized complications. As this article is focused on isolated bacteremia, the approach to treatment when there is evidence of metastatic infection will not be discussed [11].

CoNS, particularly *Staphylococcus epidermidis*, have demonstrated antimicrobial resistance to penicillin, vancomycin, and linezolid. Resistance mechanisms to methicillin and linezolid are mediated by the mecA gene and the cfr gene, respectively [12,17]. The mechanisms behind CoNS and *Staphylococcus aureus *resistance to glycopeptides, such as vancomycin, are still under investigation [18]. Given the possibility of resistance when encountering an infection with these species increases when specific testing isn’t available (e.g., a gene probe for mecA), methicillin resistance should be assumed (Table 2).

**Table 2:** Management of coagulase-negative Staphylococci bacteremia Source: [10,12,14,16,18] CoNS: Coagulase-negative Staphylococci

Pathogen	IV therapy options	Oral therapy options (if clinical criteria are met)
CoNS methicillin-sensitive strains	Flucloxacillin 2g IV every 6h; oxacillin 2g IV every 4h alternatively: vancomycin 15-20mg/kg IV every 8-12 h; cefazolin 2g IV every 6-8 hours	Trimethoprim-sulfamethoxazole 160/800mg every 12h; clindamycin 300-450mg every six hours; doxycycline 100mg every 12 hours and fluoroquinolones
CoNS methicillin-resistant strains	Vancomycin 15-20mg/kg IV every 8-12h; when resistant to vancomycin, linezolid 600mg PO/IV every 12h; daptomycin 6mg/kg IV every 24h	Trimethoprim-sulfamethoxazole 160/800mg every 12h; linezolid 600mg every 12h

Oral therapy: Oral antibiotic therapy may be used to complete treatment following an initial course of parenteral therapy. Antibiotic selection should be based on antibiotic susceptibility testing. Oral options include fluoroquinolones, trimethoprim-sulfamethoxazole 160/800mg every 12 hours, doxycycline 100mg every 12 hours, or clindamycin 300-450mg every six hours, tailored to susceptibility for uncomplicated cases [14,16].

Duration: For isolated CoNS bасterеmia, the duration depends on individual clinical circumstances. For simple CoNS bacteremia, there is evidence that suggests that it might be reasonable not to use antibiotics or use a course of maximum three days of therapy. Uncomplicated CoNS bacteremia should be treated with a five-day course of antibiotics. At least 5-7 days of IV therapy are required [11,14-16].

Enterococcus Species

Enterococcal bacteremia (EB) is a challenging condition with mortality rates reaching up to 20%. It is often complicated by antimicrobial resistance and the potential development of IE, further complicating treatment. *Enterococci*, particularly *Enterococcus faecalis *and *Enterococcus faecium,* are among the leading causes of nosocomial bloodstream infections, accounting for 10-15% of all hospital-acquired bacteremia cases. The prevalence and resistance profiles of these pathogens play a crucial role in the management of EB. *E. faecalis *accounts for 80-90% of enterococcal infections and is commonly linked to genitourinary abnormalities.* E. faecium*, though less common, is more frequently associated with multidrug resistance, including resistance to vancomycin (VRE), complicating treatment strategies. Vancomycin resistance in *E. faecium *ranges from 30 to 50% in hospital settings [19-21].

These pathogens are particularly problematic in patients with underlying comorbidities. Enterococcal infections are more likely to affect individuals with conditions such as liver cirrhosis (prevalence of 15-25% in affected populations). Advanced age, prolonged hospitalization, and exposure to broad-spectrum antibiotics are significant risk factors for enterococcal colonization. Immunosuppression and malignancies also contribute to the risk of developing EB. The sources of Enterococcal infections vary, but the gastrointestinal and genitourinary tracts are the most common reservoirs. The gastrointestinal tract is responsible for 41% of cases, often involving *E. faecium *and the genitourinary tract contributes to 27% of infections. Endovascular sources account for 15%, particularly in patients with central venous catheters or prosthetic valves and skin and soft tissue infections make up approximately 5% of cases [19-23].

Complications of enterococcal bacteremia are substantial, with IE occurring in up to 26% of *E. faecalis *bacteremia cases. Mortality rates in EB can range from 15 to 20%, with significantly higher rates in multidrug-resistant strains and immunosuppressed patients. The combination of resistance, comorbidities, and complications makes enterococcal bacteremia particularly difficult to treat (Table 3) [20-23].

**Table 3:** Management of Enterococcal bacteremia Source: [5,20-24] IV: Intravenous; PO: per os; VRE: vancomycin-resistant strains

Pathogen	IV therapy options	Oral therapy options
Enterococcus faecalis	First-line therapy: Ampicillin 2g IV every 6h. Vancomycin-susceptible strains: Vancomycin 15-20mg/kg IV every 8-12h, if 𝛃-lactam agents are contraindicated. VRE: Linezolid 600mg IV every 12h or daptomycin 10-12 mg/kg IV every 24h, potentially combined with ꞵ-lactam (ampicillin, ceftaroline, ertapenem).	Not enough data available
Enterococcus faecium	First-line therapy: Vancomycin for susceptible strains. VRE: Linezolid, daptomycin, or combination therapies (e.g., daptomycin plus ceftaroline) may be required for synergy.	

Follow-up BCs are important to ensure microbiological clearance, typically observed within 48-72 hours of starting appropriate therapy. Source control measures, such as catheter removal or abscess drainage, are critical and have a direct impact on survival. Effective management relies on accurate species identification, understanding resistance patterns, and assessing the severity of the infection. Infectious disease consultation is highly recommended, as it has been shown to increase adherence to evidence-based treatment guidelines and reduce mortality by up to 30%. Routine echocardiography is essential to ensure the timely detection of complications such as IE [1,19,20,21].

Oral therapy: There are no studies demonstrating the safety of transitioning to oral therapy in cases of uncomplicated enterococcal bacteremia. However, studies on complicated bacteremia suggest that switching to oral combination therapies, such as amoxicillin with moxifloxacin, amoxicillin with rifampicin, linezolid with rifampicin, or linezolid with moxifloxacin, has been shown to be safe. Additional studies are required to further validate its use in uncomplicated bacteremia [5]. 

Duration: Traditionally 7 to 14-day courses of monotherapy are recommended to treat uncomplicated enterococcal bacteremia. Treatment durations and optimal combinations remain poorly defined for VRE. Studies show that patients who receive short-course treatment (5-8 days) have outcomes similar to those who receive long-course treatment (12-23.5 days). The 30-day mortality rates were similar between the two groups. However, the duration of hospitalization (both overall and after the onset of bacteremia) was significantly shorter in the short-course treatment group [19,21,24]. However, the optimal duration for treating VRE and complicated enterococcal bacteremia remains poorly defined and should be guided by clinical response, resistance patterns, and the presence of complications [21,24].

Gram-negative organisms

Gram-negative organisms are a diverse group of bacteria responsible for infections ranging from mild to severe. Their distinctive cell wall structure, which includes an outer membrane with lipopolysaccharides (LPS), generally makes them more resistant to antibiotics than Gram-positive bacteria. Common pathogens like *Escherichia coli, Klebsiella pneumoniae, Pseudomonas aeruginosa* and *Enterobacter spp.* cause infections such as UTIs, pneumonia, sepsis, and intra-abdominal infections. These bacteria can develop resistance mechanisms, including beta-lactamase production, complicating treatment. Identifying and controlling the source of infection is essential for effective treatment. Tailor antibiotic therapy whenever possible [22,24].

*Pseudomonas aeruginosa *and *Klebsiella pneumoniae *are significant pathogens associated with high morbidity and mortality in bloodstream infections. The Infectious Diseases Society of America (IDSA) and the American Thoracic Society (ATS) guidelines emphasize the importance of appropriate initial antimicrobial treatment for *Pseudomonas aeruginosa* bloodstream infections due to their association with high mortality rates [1].

Studies have shown that timely and appropriate antibiotic therapy is crucial for *Klebsiella pneumoniae* bloodstream infections, particularly those caused by carbapenemase-producing strains, to reduce mortality [1,22-24].

Given the potential severity of infections caused by these organisms, even a single positive BC warrants prompt initiation of appropriate antimicrobial therapy to improve patient outcomes. Follow-up BCs have limited value once the source is controlled.

Traditionally, the recommended duration has been 10-14 days. Studies provide strong evidence supporting the use of a seven-day antibiotic regimen for patients with uncomplicated Gram-negative bacteremia who are stable and have no complicating factors. Regardless of the duration, at least two days of IV antibiotics should be administered before transitioning to oral therapy [2,19].

Shortening the duration of therapy reduces the risk of antibiotic resistance and side effects, without negatively affecting patient outcomes. These findings are in line with current recommendations in some clinical guidelines advocating for short-course antibiotic therapy for uncomplicated bacteremia [19]. However, clinicians should always assess individual patient circumstances, including any underlying conditions or complicating factors, before determining the optimal duration of therapy. The treatment of specific Gram-negative pathogens will now be discussed.

Enterobacterales

The* Enterobacterales *order includes a diverse group of Gram-negative bacteria such as *Escherichia coli, Klebsiella, Enterobacter, Proteus, Serratia and Citrobacter.* These are often associated with infections of the urinary and gastrointestinal tracts, which can complicate with bacteremia (Table 4). The increasing display exhibition of multidrug resistance has become a growing public health concern and led to higher mortality rates due to inadequate treatment. The primary mechanism of resistance in these organisms is associated with the synthesis of β-lactamases, which are classified into different groups, such as extended-spectrum β-lactamases (ESBL), AmpC β-lactamases, and those that confer carbapenem resistance, including* Klebsiella pneumoniae *carbapenemase (KPC), metallo-β-lactamases (MBL), such as New Delhi Metallo-β-Lactamase (NDM), and oxacillinases (OXA β-lactamases) [25-28]. 

**Table 4:** Management of Enterobacterales bacteremia Source: [25-28] ESBL: Extended-spectrum β-lactamases; KPC: Klebsiella pneumoniae carbapenemase; IV: intravenous; OXA: oxacillinases; PO: *per os*

Pathogen	IV therapy options	Oral therapy options (if clinical criteria are met)
Enterobacterales susceptible Strains	Ceftriaxone 2g IV every 24h; piperacillin-tazobactam 4.5g IV every 6h.	Trimethoprim-sulfamethoxazole 160/800mg every 12h and fluoroquinolones (e.g., ciprofloxacin 750mg PO every 12h).
Enterobacterales ESBL-producing Strains	Carbapenems (e.g., meropenem 1g IV every 8h).	Fluoroquinolones (e.g., ciprofloxacin 750mg PO every 12h)
Enterobacterales AmpC 𝛃-lactamases-producing strains	Cefepime 2g IV every 12h; carbapenems (e.g., Meropenem 1g IV every 8h).	Fluoroquinolones (e.g., ciprofloxacin 750mg PO every 12h); trimethoprim-sulfamethoxazole 160/800mg every 12h.
Enterobacterales KPC-producing Strains	Ceftazidime-avibactam 2.5g IV every 8h; meropenem-vaborbactam 4g IV every 8h; imipenem-cilastatin-relebactam 1.25g IV every 6h.	Not enough data available
Enterobacterales metallo-𝛃-lactamases-producing strains	Aztreonam 2g IV every 6h and ceftazidime-avibactam 2.5g IV every 8h; association Meropenem-vaborbactam 4g IV every 8h; cefiderocol 2g IV over 3h every 8h.	Not enough data available
Enterobacterales OXA β-lactamases producing strains	Ceftazidime-avibactam 2000 mg/500 mg every 8h Ceftaroline-avibactam 600/600mg every 8h; cefiderocol (2g IV every 8h).	Not enough data available

Oral therapy: Oral therapy is proven to be effective for the treatment of *Enterobacterales* bloodstream infections if there is proper source control and favorable clinical response to initial IV therapy. If oral agents are available (usually for *Enterobacterales* producing ESBL or AmpC, but not for oxacilinases, metallo-β-lactamases or other carbapenem-resistant strains), switching to oral therapy after 2 days of IV therapy can be considered. It is recommended the use of fluoroquinolones or trimethoprim/sulfamethoxazole for their high bioavailability, if there is in vitro susceptibility [27,29].

Duration: The latest literature suggests that seven-day antibiotic courses appear to be the preferred strategy for the treatment of bloodstream infections caused by *Enterobacterales*, provided adequate source control is ensured. This approach seems to achieve similar rates of clinical resolution vs. symptomatic recurrence when compared to 14-day courses, while demonstrating fewer therapy-related adverse events, both in previously healthy individuals and in patients with greater severity, such as immunosuppressed individuals or cases of severe infection [28].

Pseudomonas aeruginosa

A notorious pathogen in immunocompromised individuals and hospital-acquired infections, *Pseudomonas aeruginosa *has a remarkable ability to develop resistance to multiple antibiotics. This resistance, coupled with its ability to cause invasive infections, underscores the need for aggressive and targeted therapy. Management strategies often involve antipseudomonal agents and understanding when to use combination therapy can be life-saving. Current evidence does not support the use of combination therapy over monotherapy for treating *Pseudomonas aeruginosa *bacteremia when susceptibility data are available. Studies show no significant difference in cure rates or mortality between beta-lactam monotherapy and combination therapy. However, aminoglycosides should generally not be used as monotherapy. Research indicates that aminoglycoside monotherapy is associated with worse outcomes compared to beta-lactam monotherapy. For infections caused by *Pseudomonas* isolates resistant to both beta-lactams and anti-pseudomonal fluoroquinolones, consider consulting an infectious diseases specialist to guide management (Table 5) [19,29,30].

**Table 5:** Management of Pseudomonas aeruginosa bacteremia Source: [19,29,30] ESBL: Extended-spectrum β-lactamases; KPC: Klebsiella pneumoniae carbapenemase; IV: intravenous; OXA: oxacillinases; PO: per os

Pathogen	IV therapy options	Oral therapy options (if clinical criteria are met)
Pseudomonas aeruginosa-susceptible strains	First-line therapy: Piperacillin-tazobactam 4.5g IV every 6h; ceftazidime 2g IV every 8h; cefepime 2g IV every 8h. Alternative: Meropenem 2g IV every 8h can be used in selected cases.	Ciprofloxacin 750mg PO every 12h; levofloxacin 750mg PO every 24h if allowable by the susceptibility profile.
Pseudomonas aeruginosa AmpC-producing strains	Meropenem 2g IV every 8h; Ceftolozane-tazobactam 1.5g IV every 8h; ciprofloxacin 400mg IV every 8h	Ciprofloxacin 750mg PO every 12 hours, if strain susceptible
Pseudomonas aeruginosa carbapenem-resistant strains	Ceftazidime-avibactam 2.5g IV every 8h; Imipenem-cilastin-relebactam 1.25g IV every 6h	Not enough data available
Pseudomonas aeruginosa Metallocarbapenemase-producing strains	First-line therapy: Ceftazidime-avibactam 2.5g IV every 8h plus Aztreonam 2g every 6h; cefiderocol 2g IV every 8h Alternative: Ceftolozane-tazobactam, ceftazidime-avibactam, or cefiderocol.	Ñot enough data available

Oral therapy: A minimum of five days of IV therapy is required. In selected patients, switching to oral therapy may be considered when dealing with susceptible or ESBL/AmpC-producing strains, after susceptibility to fluoroquinolones is tested. There is still no oral regime suggested for successful treatment of other resistant strains. [29,30].

Duration: The duration of treatment is 8-10 days. Studies show that using longer courses of 14-17 days doesn’t have an impact on the patient outcomes [19,29,30].

Consideration of the transition to oral therapy

The transition from IV to oral antibiotic therapy represents a significant advancement in the management of bacteremia, particularly for low-risk patients. This approach is supported by clinical evidence, including the SABATO trial, which demonstrated non-inferiority of oral regimens compared to prolonged IV therapy in patients with low-risk *Staphylococcus aureus *bacteremia. Transitioning to oral therapy not only reduces the burden on healthcare systems but also improves patient outcomes when applied judiciously [3,5].

Criteria for transitioning from IV to oral therapy include clinical stability, effective source control, intact oral absorption, availability of appropriate antibiotics, and patient adherence potential. Clinical stability requires resolution of fever for at least 48-72 hours, stable hemodynamic parameters without vasopressors, and noticeable improvement in infection-related symptoms, such as reduced markers of inflammation like C-reactive protein or procalcitonin. Source control is equally critical, ensuring that primary infection sites are managed effectively through abscess drainage, removal of infected prosthetic devices or catheters, or debridement of infected tissues [3,5,8]. 

Oral intake capabilities must be intact, necessitating effective oral absorption. Conditions like nausea, vomiting, or malabsorption should be addressed before transitioning to oral antibiotics. Additionally, the availability of oral agents with high bioavailability and sufficient activity against the causative pathogen is vital. Examples of effective oral antibiotics include fluoroquinolones for Gram-negative infections, Trimethoprim-sulfamethoxazole or clindamycin for Gram-positive infections, and linezolid for certain resistant organisms. Finally, psychosocial and socio-economical evaluation is essential, because it allows physicians to establish the likelihood of therapeutic adherence, enabling interventions (patient and family education, deployment of community resources, etc.) that might increase the odds of a successful treatment [2,3,8].

Transitioning to oral therapy offers multiple benefits. It significantly reduces the length of hospital stays, minimizing patient exposure to nosocomial infections and freeing up healthcare resources. Oral therapy also eliminates the risk of catheter-associated bloodstream infections and thrombophlebitis, which are common complications of prolonged IV therapy. Additionally, oral regimens allow greater patient mobility, improving psychological well-being and overall quality of life by reducing dependence on hospital-based care [2,3,6].

Lower healthcare costs are another major advantage, as oral therapy reduces resource utilization, including hospital bed occupancy and the preparation of IV medications. For patients, the convenience of oral therapy eliminates the need for home infusion services or frequent hospital visits, thereby enhancing treatment adherence and overall satisfaction [2,3,6].

The transition from IV to oral antibiotic therapy represents a significant advancement in the management of bacteremia, particularly for low-risk patients. It is supported by clinical evidence, including the SABATO trial, which demonstrated non-inferiority of oral regimens in patients with low-risk *Staphylococcus aureus bacteremia*. Similarly, studies on Gram-negative bacteremia have demonstrated that carefully selected patients can safely transition to oral regimens without increased risk of treatment failure [2,3,5,9,29].

Considerations of the duration of therapy

Research indicates that shorter courses of antibiotics are often as effective as longer ones and can reduce side effects and the risk of antibiotic resistance. Determining the appropriate duration of therapy is critical for ensuring treatment efficacy while minimizing the risks of overuse. For SAB, uncomplicated cases may require a minimum of 14 days of therapy, with the duration calculated from the first negative BC [11]. In uncomplicated bacteremia, a seven-day course for Gram-negative bacteremia is recommended when source control has been achieved and the patient is clinically stable [14]. The rationale for shorter courses is supported by robust evidence showing non-inferiority in selected cases, providing benefits such as reduced antimicrobial resistance, fewer adverse drug events, and lower treatment costs. Ultimately, therapy duration should be individualized based on the pathogen type, infection site, and patient-specific factors such as immune status and comorbidities. This tailored approach ensures that each patient receives the most appropriate and effective treatment for their condition [2,5,19,29].

Discussion

The findings underscore a paradigm shift in bacteremia management, moving away from traditional prolonged IV antibiotic courses toward more individualized approaches. The adoption of shorter antibiotic regimens and early oral transitions, supported by stringent criteria, offers multiple benefits, including reduced hospital stays, lower healthcare costs, and improved patient quality of life [2,3,5,29].

However, these approaches are not without challenges. Resistance patterns, patient non-adherence, and the need for precise clinical judgment emphasize the importance of multidisciplinary care involving infectious disease specialists. Additionally, ongoing research is necessary to refine guidelines and address gaps in evidence, particularly for multidrug-resistant infections [13,14].

Finally, Table 6 provides a comprehensive overview of the treatment strategies for bacteremia caused by key pathogens, including Staphylococcus aureus, Coagulase-negative Staphylococci, Enterococcus species, Enterobacterales, and Pseudomonas aeruginosa. The table highlights critical aspects of therapy, such as the recommended duration and considerations for switching to oral treatment, offering valuable guidance for optimizing patient management.

**Table 6:** Pathogen-specific approaches to facilitate a reduced antibiotic duration and early oral therapy transition Source: [2,3,5,6,10,16,19,20,25,29,30] ATB: Antibiotic

Agent of uncomplicated bacteremia	Minimum ATB course	Minimum IV duration	Oral switch
Staphylococcus aureus	14 days	5 - 7 days	✔
*Staphylococcus *coagulase negative	0-3 or 5 days (algorithm based)	5 days	✔
Enterococcus	7 days	-	-
Enterobacterales	7 days	2 days	✔
Pseudomonas aeruginosa	8 - 10 days	5 days	✔

## Conclusions

Advancements in bacteremia treatment highlight the potential for optimizing care through evidence-based practices. Shorter courses and oral therapy transitions are safe and effective for carefully selected patients, provided clinical stability and appropriate criteria are met. These strategies not only improve patient outcomes but also support antimicrobial stewardship by reducing unnecessary antibiotic use.

Future research should focus on further defining criteria for therapy transitions, exploring novel antibiotics for resistant pathogens, and assessing the long-term impact of these approaches on resistance trends and patient quality of care.
